# Education in diabetes mellitus with focus on social support: longitudinal study

**DOI:** 10.1186/1758-5996-7-S1-A183

**Published:** 2015-11-11

**Authors:** Danielle dos Santos Gomides, Lilian Cristiane Gomes Villas-Boas, Jonas Bodini Alonso, Maria Cristina Foss-Freitas, Milton Cesar Foss, Ana Emilia Pace

**Affiliations:** 1 Escola de Enfermagem de Ribeirão Preto, Ribeirão Preto, Brazil

## Background

Education in diabetes mellitus is an ongoing process to promote/encourage the acquisition of knowledge on self-care skills and control of the disease and this process can be facilitated by social support from family. The literature shows the benefits of educational interventions for people with diabetes mellitus, however, there are few studies on its long-term effects. Objective: To assess the effectiveness of educational interventions focusing on social support after two yrs. of its completion.

## Method

Quantitative/longitudinal study whose data before (T0) and after 12 months (T12) came from a clinical trial in which participants were given educational interventions through the “Conversation Maps in Diabetes”, tool and there was involvement of the family through an Intervention Group, done by phone calls. The third collection (T36) of data was from the population (N=164) that participated of clinical trial. The variables of interest was the knowledge of the disease/care, evaluated by “Diabetes Knowledge Scale” (DKN-A) validated in Brazil, and glycemic control assessed by glycated hemoglobin value obtained in electronic medical records. In the statistical analysis we used the nonparametric ANOVA for repeated measures.

## Results

**Figure 1:**
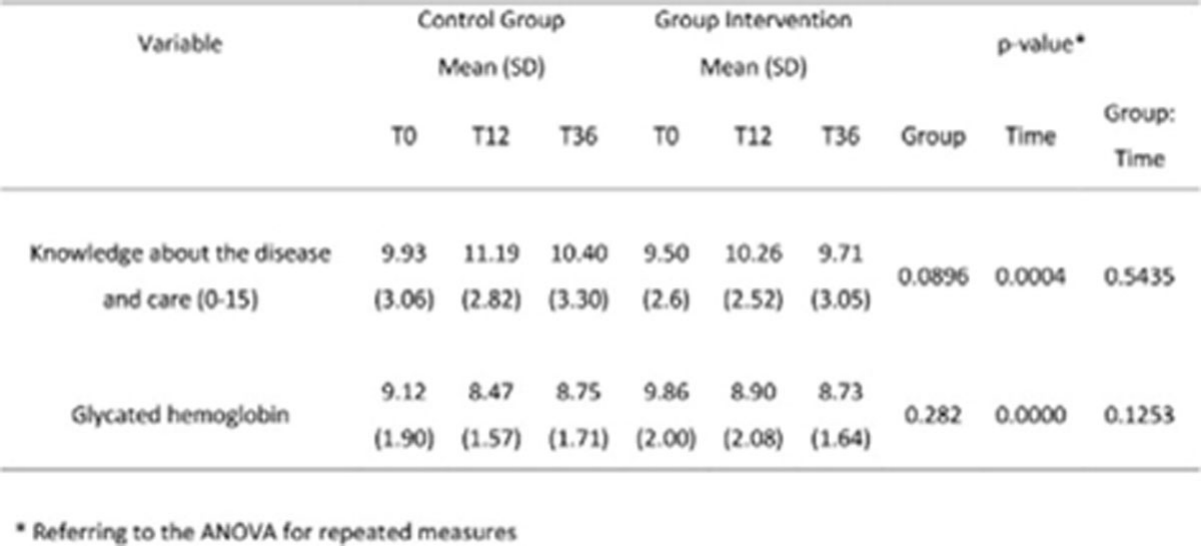
Knowledge about the disease and glycated hemoglobin. Ribeirão Preto, 2015.

Of the 164 participants (82 of the Intervention and 82 of the Control Group), 95 (50 and 45 respectively) were interviewed. There were differences in the values of knowledge (p-value=0.0004) and glycated hemoglobin (p-value=0.0001) in the studied time (Figure 1).

## Conclusion

The highest score of knowledge was at T12 in both groups, with a reduction in T36, reiterating the importance of education as an ongoing process. As for glycemic control, family social support seems to have influenced the maintenance of results.
